# Left Ventricular Diastolic Dysfunction in the Intensive Care Unit: Trends and Perspectives

**DOI:** 10.1155/2012/964158

**Published:** 2012-05-14

**Authors:** Lewis Ari Eisen, Pericles Davlouros, Dimitrios Karakitsos

**Affiliations:** ^1^Jay B. Langner Critical Care Service, Division of Critical Care Medicine, Department of Medicine, Montefiore Medical Center, Albert Einstein College of Medicine, Bronx, NY 10467, USA; ^2^Department of Cardiology, University Hospital of Rio, 26504 Patra, Greece; ^3^Critical Care Unit, General State Hospital of Athens, 11437 Athens, Greece

## Abstract

Heart failure with a normal or nearly normal left ventricular (LV) ejection fraction (HFNEF) may represent more than 50% of heart failure cases. Although HFNEF is being increasingly recognized, there is a relative lack of information regarding its incidence and prognostic implications in intensive care unit (ICU) patients. In the ICU, many factors related to patient's history, or applied therapies, may induce or aggravate LV diastolic dysfunction. This may impact on patients' morbidity and mortality. This paper discusses methods for assessing LV diastolic function and the feasibility of their implementation for diagnosing HFNEF in the ICU.

## 1. Introduction

Diastolic heart failure (DHF) has been described since 1998 [1]. At that time, it was thought to be less frequent than systolic heart failure (SHF) and have a better prognosis [2]. Nowadays, DHF is known to account for more than 50% of all heart failure patients, with a similar prognosis to SHF [3–5]. Diastolic left ventricular (LV) dysfunction is associated with slow LV relaxation and increased LV stiffness [6]. Many factors can result in DHF such as ventricular hypertrophy, myocardial fibrosis, infiltrative disease, pericardial constrictive disorders, right ventricular (RV) alterations due to a variety of causes, advanced age, hypoxia, and acidosis, but most commonly coronary artery disease (CAD) [3, 4]. Therefore, DHF may coexist with SHF, leading to the formulation of a “single syndrome” hypothesis, which postulates that diastolic LV dysfunction is actually a precursor of SHF and is due to increased interstitial deposition of collagen and modified matricellular proteins [3]. For this reason, experts propose the term heart failure with normal ejection fraction (HFNEF) instead of DHF, to indicate that HFNEF could be a precursor to heart failure with reduced LVEF [3, 4].

Diagnosis of HFNEF requires the presence of heart failure symptoms and signs, with normal or mildly abnormal LVEF, (LVEF >50% and LV end-diastolic volume index <97 mL/m^2^), and evidence of LV diastolic dysfunction [3, 4]. The latter is associated with slow LV relaxation, increased LV stiffness, reduced ventricular compliance, and increased LV filling pressure. Thus, it can be diagnosed invasively by means of right heart catheterization (LV end-diastolic pressure >16 mmHg or mean pulmonary capillary wedge pressure >12 mmHg). Alternatively, echocardiography may be used for noninvasive assessment of diastolic dysfunction. Newer echocardiographic techniques like tissue Doppler (TD) have provided indices of LV diastolic dysfunction like the TD derived *E*/*E*′ > 15 (early transmitral flow velocity/early TD diastolic lengthening velocity). If this ratio is inconclusive, (15 >  *E*/*E*′ > 8), then additional echocardiographic information relevant to LV diastolic dysfunction can be derived by Doppler interrogation of mitral valve or pulmonary veins or left atrial volume index. Finally, elevated levels of plasma natriuretic peptides may aid in the diagnosis of DHF [3, 4, 7].

Nagueh et al. provide a simple recommendation for grading LV diastolic dysfunction by using pulsed Doppler at the mitral valve and at the mitral annulus [7]. This is briefly reviewed here but can be seen in full detail in the original paper [7]. In all forms of diastolic dysfunction left atrial volume should be greater than 34 mL/m^2^. In mild (Grade I) diastolic dysfunction, the mitral E/A ratio is <0.8, and the deceleration time of the E wave (DT) is >200 ms. In moderate (Grade II) diastolic dysfunction, the mitral E/A ratio is 0.8–1.5 and DT is 160–200 ms. In severe (Grade III) diastolic dysfunction, the mitral E/A ratio is ≥2 and DT<160 ms. The severity of diastolic dysfunction predicts mortality in longitudinal followup of outpatients [8].

While useful, there are several problems with this classification. A reduced mitral E/A ratio may be seen in hypovolemia. The majority of people >60 years old have E/A ratios <1 and DT >200 ms and in the absence of other indications of cardiac disease should be considered normal [7]. Trained athletes may have enlarged left atrial volumes. Doppler measurements can show individual variability and can vary with changes in preload, afterload, and sympathetic tone. As long as the operator is aware of these factors, the grading system can be very helpful for clinical practice as it is a simple method of communicating important information. Additionally, it can be valuable for research trials as it can be used to compare different populations in a standardized fashion.

## 2. LV Diastolic Dysfunction in the Intensive Care Unit (ICU)

In the ICU, there are many scenarios where factors influencing LV relaxation, diastolic distensibility, and filling pressures coexist. These factors may be linked to underlying disorders (CAD, arrhythmia, valvular dysfunction, pericardial disease, sepsis, and hypoxia), to patients' history (age, hypertension, diabetes mellitus, and chronic renal failure), or to applied therapies (volume resuscitation and positive end-expiratory pressure (PEEP)). Despite the fact that HFNEF has been increasingly identified, its incidence and impact on prognosis in critically ill patients in the ICU remain uncertain. The ICU-specific literature is reviewed below, but due to its sparse nature, extrapolations sometimes have to be made from the non-ICU cardiology literature. This is not only due to lack of extensive research in this setting, but also due to practical differences in patient populations. The clinical signs and symptoms of HF required for the diagnosis of HFNEF may be difficult to recognize in the ICU patient [3]. The presence of normal or mildly abnormal systolic LV function, which constitutes the second criterion for the diagnosis of HFNEF, is easily identified by echocardiography with the generally accepted definition of normal or mildly abnormal LVEF being >50% [3, 4]. Additionally, normal or mildly abnormal LVEF depends on the time elapsed between the clinical heart failure episode and the echocardiographic examination. Thus, it is recommended that information on LV systolic function be obtained within 72 h following the heart failure episode. In ICU patients, echocardiographic examination should be done promptly, once signs of possible heart failure are present.

The typical patient seen in the MICU or surgical ICU differs from a CCU patient. In the CCU, diastolic dysfunction is often seen in the context of coronary artery disease, valvular disease, or arrhythmias. While these may be present in the MICU or SICU patient, there will be a higher percentage of sepsis, renal failure, and hypoxemia. In the MICU or SICU patient there will be a higher incidence of noncardiac comorbidity; so differentiating cardiac from noncardiac causes of dyspnea is of great importance. Finally, there will be a higher percentage of applied therapies that may affect diastolic heart function such as fluid resuscitation and positive pressure ventilation.

Importantly the methods used to assess LV relaxation, diastolic distensibility, stiffness, and filling pressure suffer from many drawbacks in the ICU setting. Hence, even invasive measurements may produce inconclusive results, and finding a clinically hypovolemic patient with “normal” pulmonary artery catheter wedge pressure (PCWP) or a normovolemic patient with an elevated PCWP is not uncommon. Doppler indices of diastolic dysfunction only moderately correlate with invasive parameters [9, 10]. Also, echocardiography, which has been widely applied for providing diagnostic and monitoring solutions in patients with HFNEF, carries well-known flaws [3, 4, 7], and some of the newer echocardiographic techniques (i.e., strain, strain rate, etc.) may be difficult to conduct in the ICU.

While critical care ultrasound is a growing field, the nature of ICU practice leads to several limitations to ultrasound use. Many ICU patients are receiving mechanical ventilation which may impede imaging of the heart. ICU patients sometimes cannot be positioned adequately for all cardiac views. Surgical wounds, dressings, subcutaneous emphysema, tubes, and foreign devices may obstruct views [11]. The (at least partial) failure rate of TTE in the ICU setting has been reported to be between 30 and 40% in older studies [12, 13]. Contrast echocardiography or harmonic imaging can help in some cases [14]. Also, many of these limitations can be overcome with the use of TEE when clinically indicated. With proper training, noncardiologist intensivists can perform adequate TEE examinations [15, 16].

The presence of concentric LV remodeling may have important implications for the diagnosis of HFNEF, and an increased LV wall mass index may provide sufficient evidence for the diagnosis of HFNEF when TD yields nonconclusive results or when plasma levels of natriuretic peptides are elevated [3]. The latter, (Atrial natriuretic peptide (ANP) and brain natriuretic peptide (BNP)) are produced by atrial and ventricular myocardial cells in response to an increase of atrial or ventricular diastolic stretch and mediate natriuresis, vasodilation, and improved LV relaxation. Their diagnostic accuracy for HF has been established, and combination with TD-derived *E*/*E *′ ratio may prove an extremely valuable tool in the ICU setting for diagnosis of HFNEF [3, 4]. Finally, left atrial enlargement and/or evidence of atrial fibrillation are considered adjunctive evidence for the diagnosis of HFNEF [3, 4].

Recent studies have linked the presence of diastolic dysfunction to weaning failure in the critically ill [17–19]. Papanikolaou et al. have studied a small series of critical care patients with preserved LV systolic function and reported that weaning failure was related not only to grade III but also to grade I diastolic dysfunction [17], while Caille et al. reported similar results in 117 unselected patients; however diastolic dysfunction was often associated with systolic dysfunction in the latter series [18]. Lamia et al. discovered that an increase in LV filling pressures related to spontaneous breathing trials (SBTs) was predictive of weaning failure in a highly selected population (patients with two preceding failed SBTs, and approximately 20% of them had a decreased LVEF) [19]. In outpatients, relaxation impairment of the LV can be unmasked by performing Doppler echocardiography during exercise [20]. In the ICU, weaning trials can be considered as exercise due to increments in respiratory and cardiovascular load and in oxygen demand [17–21]. Hence, weaning may reveal subtle diastolic dysfunction in the ICU.

Another issue associated with LV diastolic dysfunction, which is largely unstudied, is its possible relation to acute loading and unloading conditions of the LV commonly observed in the ICU. An LV with diastolic dysfunction is considered in cardiodynamic terms volume sensitive hence exploring those echocardiographic indices as aids to guide fluid loading or unloading is of great clinical interest. Tissue Doppler indices might be extremely useful in this regard, as *E*′ can be conceptualized as the amount of blood entering the LV during early filling, whereas *E* represents the gradient necessary to make this blood enter the LV. Therefore, a high *E*/*E*′ represents a high gradient for a low shift in volume [3]. Additionally, echocardiography may be used with adjunctive lung ultrasound examination, as the latter may identify alveolar-interstitial syndrome by evaluating lung-rocket artifacts (B-lines) that may provide additional information about lung water [22, 23].

The concept of isolated diastolic dysfunction is becoming a new trend in cardiodynamic analysis; however, as previously mentioned, cases which have been characterized as isolated diastolic dysfunction may well exhibit “subtle” systolic dysfunction [3, 4]. This energy interaction between systole and diastole will surely produce further pathophysiologic debate and might also lead towards new concepts in the development of ventricular assist devices [24]. In theory, alterations in LV stiffness that relate to diastolic dysfunction might be linked as well to changes in the three-dimensional (3D) systolic twisting and diastolic untwisting of the LV. However, the effects of load and inotropic state on LV systolic twist and diastolic untwist in human subjects remain to be studied [25]. The interaction of altered 3D ventricular geometry with the formatting vortices observed in the LV by modern magnetic resonance imaging techniques and complex fluid-structure numerical models may hold the key to the pathophysiologic development of diastolic dysfunction [26, 27]. Yet again the latter may represent another example of phenotypic plasticity, the capacity of a genotype to exhibit a range of phenotypes in response to environmental variations [28], reflecting a physiologic adaptation of the ventricle to altered myocardial cell structure and disturbed flow patterns in states of cardiovascular disease [29, 30].

Furthermore, LV tolerance to fluid loading might be better monitored by ultrasound in common scenarios such as the resuscitation of septic shock. In the latter, experimental models and clinical studies have previously reported that apart from systolic dysfunction, alterations in LV stiffness and various grades of diastolic dysfunction may exist [31–37]. In such patients, diastolic dysfunction seems to be an independent predictor of mortality [31].

Consideration should be given to the issue of training of intensivists in echocardiographic analyses of patients with diastolic dysfunction. In the United States, only 55% of critical care fellowship programs provide training in echocardiography [38]. How many of these provide training in analysis of diastolic dysfunction is unknown as this is not recommended by recent guidelines [39, 40]. In our experience, the extra training time required to perform basic analysis of diastolic dysfunction is not excessive. However, depending on the skill of the examiner and the clinical scenario, advanced consultation from expert-level echocardiographers may be required.

Despite the fact that we have still much to learn about many of the above-mentioned mechanisms, by integrating sophisticated “functional cardiac imaging” techniques with current research our clinical understanding of the specificities encountered in critical care patients who may present with LV diastolic dysfunction will be improved. Knowledge of diastolic dysfunction should not be considered a sophisticated approach designated only for cardiologists but should be familiar to all intensivists.

## 3. Conclusion

Although HFNEF is being increasingly recognized, there is a relative lack of information regarding its incidence and prognostic implications in the critically ill. There may be difficulties in the implementation of criteria for the diagnosis of HFNEF in the ICU. However combination of simple echocardiographic indices of LV diastolic dysfunction like TD-derived *E*/*E*′ with other simply derived echocardiographic parameters like left atrial size, or presence of left ventricular hypertrophy with natriuretic peptides, may prove invaluable tools for studying the role of diastolic LV dysfunction in such patients.
